# Gynecologic Survivorship Tool: Development, Implementation, and Symptom Outcomes

**DOI:** 10.1200/CCI.21.00154

**Published:** 2022-03-03

**Authors:** Jeanne Carter, Nadeem R. Abu-Rustum, Sally Saban, Ling Y. Chen, Andrew J. Vickers, Amy L. Tin, Gabriela Billanti, Nicole A. Connors, Vance Broach, Carol L. Brown, Dennis S. Chi, Ginger J. Gardner, Deborah J. Goldfrank, Elizabeth L. Jewell, Mario M. Leitao, Kara C. Long Roche, Jennifer J. Mueller, Yukio Sonoda, Oliver Zivanovic

**Affiliations:** ^1^ Gynecology Service, Department of Surgery, Memorial Sloan Kettering Cancer Center, New York, NY; ^2^ Department of Psychiatry, Memorial Sloan Kettering Cancer Center, New York, NY; ^3^ Department of Psychiatry, Weill Cornell Medical College, New York, NY; ^4^ Department of Obstetrics and Gynecology, Weill Cornell Medical College, New York, NY; ^5^ Department of Epidemiology and Biostatistics, Memorial Sloan Kettering Cancer Center, New York, NY

## Abstract

To describe the development and implementation of a new digital health clinical tool (Gynecologic Survivorship Tool [GST]) for symptom management of women surgically treated for gynecologic cancer; to assess its feasibility; and to conduct a retrospective review of the data.

**MATERIALS AND METHODS:**

The GST was developed on the basis of a comprehensive review of the literature, multidisciplinary expert opinion, and feedback from women with a history of gynecologic cancer. It is composed of 17 questions addressing six main categories—gynecologic health (abnormal bleeding/pain), lymphedema, vaginal/vulvar dryness, sexual health, menopause (hot flushes/sleep difficulties), and bowel/urinary issues. An electronic version using the Memorial Sloan Kettering Cancer Center Engage platform was piloted in two clinics for patients with endometrial or cervical cancer. Health information was generated into clinical summaries and identified concerns for actionable response. Associations of symptom and survey time point were assessed by longitudinal models using generalized estimating equations.

**RESULTS:**

From January 1, 2019, to February 29, 2020, 3,357 GST assessments were assigned to 1,405 patients, with a 71% completion rate (90% within 5 minutes). Sixty-eight percent were performed at home via a patient portal, 32% at follow-ups using a clinic iPad. The most common symptoms were bowel problems, swelling/fluid, pain during examination, vaginal or vulvar dryness, and vaginal bleeding. Implementation challenges included improving patient compliance and ensuring that reports were reviewed by all clinical teams. We developed screening e-mails detailing patients whose assessments were due, planned training sessions for multidisciplinary teams, and incorporated feedback on methods for reviewing symptoms reports.

**CONCLUSION:**

The GST demonstrated feasibility, a high completion rate, and minimal time commitment. It was an effective electronic reporting mechanism for patients, enabling the medical team to develop specific strategies for alleviating bothersome symptoms during follow-up.

## INTRODUCTION

### Background and Significance

Patients undergoing treatment for cancer often experience symptoms that persist after treatment and into survivorship. Several studies have shown that integration of patient-reported outcomes (PROs) into the management of cancer survivors is associated with improved symptom control compared with routine care.^1-3^ PROs are particularly valuable, because the information comes directly from the patient rather than a clinician or family member. Clinicians recognize the importance of symptom management but, unfortunately, are often unaware of symptoms in real time.^4,5^ Methods to routinely screen patients, capture symptom concerns, and translate this information directly into the clinical setting are needed.

**CONTEXT**

**Key Objective**
Is the development and implementation of an electronic-based survivorship tool for patients with gynecologic cancer—to collect and address patients' symptoms in real time, identify appropriate interventions, and promote patient/clinician communication—feasible and useful in a busy clinical setting?
**Knowledge Generated**
Integration of patient-reported outcomes into the management of patients with cancer is associated with improved symptom control; electronic platforms create meaningful ways for patients and clinicians to track and address postoperative symptoms virtually. The Gynecologic Survivorship Tool is readily implemented, requires minimal time commitment, and identifies actionable items and appropriate interventions.
**Relevance**
In patients with gynecologic cancer, attention to sexual health concerns early in treatment and follow-up can lead to better outcomes and quality of life. The Gynecologic Survivorship Tool is a user-friendly and effective digital tool. Such tools offer an opportunity for safe, efficient, and effective delivery of care, and should be considered as a potential investment by institutions.

The literature shows recent attempts to enhance the flow of information between patients and providers. A recent randomized controlled trial examining symptom summaries and PROs in radiation oncology clinics revealed an increased association between patient-reported symptoms on computer forms and discussions between patients and providers,^6^ with some evidence of improved symptom management. Another randomized controlled trial used a smartphone mobile app to support treatment recommendations, with the goal of fostering adherence to oral cancer therapy and symptom management.^7^ Electronic platforms in a real-world setting have high levels of acceptability^8-11^ and offer the opportunity for an intersection between health care and technology to optimize patient care.

### Objective

Our objective was to develop a feasible electronic-based survivorship tool for patients with gynecologic cancer to collect symptom concerns before follow-up and generate summaries to be used within a busy clinical setting, promoting patient/clinician communication and plans for symptom management. Here, we describe challenges in the development and implementation of this digital tool to provide effective care.

## MATERIALS AND METHODS

### Development of the Gynecologic Survivorship Tool

To develop the tool, we conducted a literature review of gynecologic survivorship issues and of existing PROs scales (Functional Assessment of Cancer Therapy [FACT], European Organization for Research and Treatment: Quality of Life Questionnaire [EORTC-QLQ-30], Gynecologic Cancer Lymphedema Questionnaire [GCLQ], Common Terminology Criteria for Adverse Events [CTCAE], and Female Sexual Distress Scale [FSDS]). A multidisciplinary group of experts (surgical, medical, and radiation oncologists; nurses; a psychologist; and an epidemiologist) evaluated items for potential inclusion and provided feedback about the content and assessment domains. The goal was to identify symptoms of concern for intervention without using extensive questionnaires, decrease patient burden, and increase patient participation. Six volunteer gynecologic cancer survivors were then given an overview of the rationale and anticipated use of the Gynecologic Survivorship Tool (GST); they provided feedback on its readability and whether all important symptoms and concerns were included. On the basis of this feedback, separate items addressing vaginal compared with vulvar concerns were added. Survivors were asked whether they would be willing to complete such a survey and whether they would prefer to do this at home or in clinic. They approved of the options to complete the survey at follow-up appointments or at home. They deemed the items to be appropriate for survivorship and appreciated the inclusion of items addressing bowel/bladder issues and sleep.

### Design

**FIG 1.**
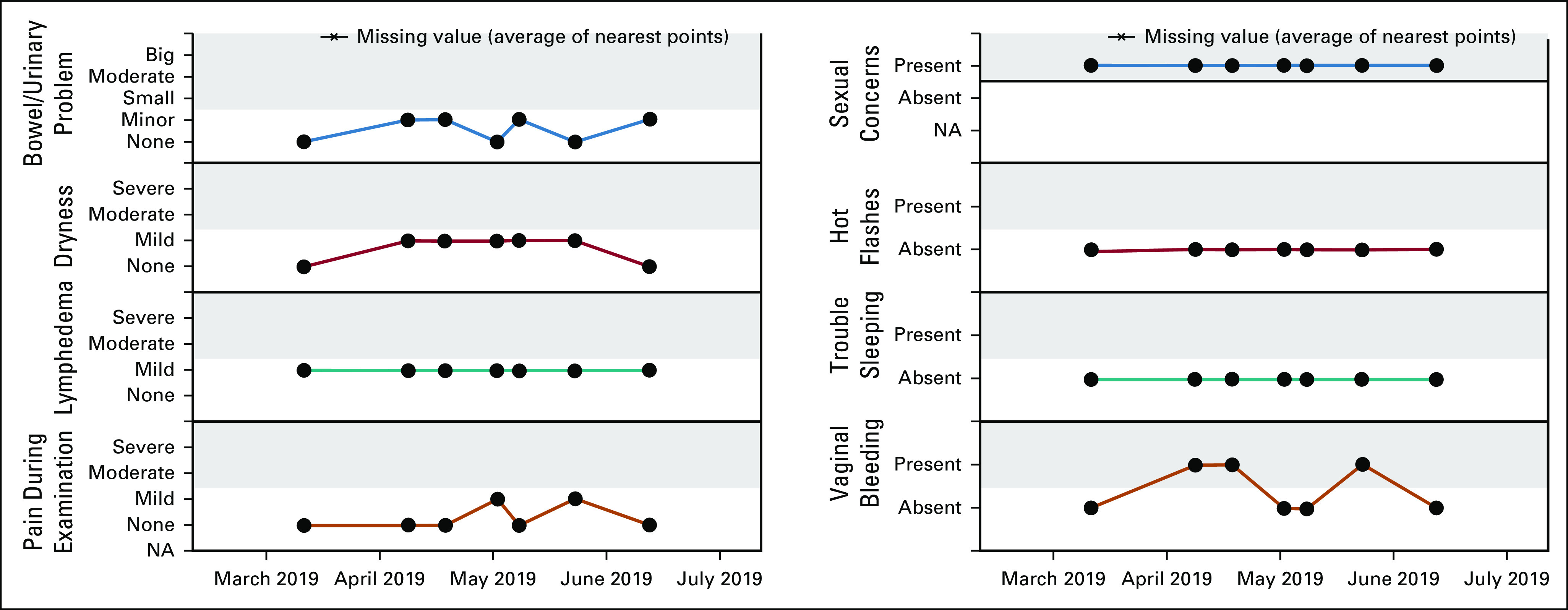
Clinical symptom summary report. NA, not available.

The tool is composed of 17 questions addressing six main categories: gynecologic health (abnormal bleeding/pain), lower-extremity lymphedema (heaviness, swelling, and numbness), vaginal/vulvar dryness, sexual activity, and sexual health concerns (libido, arousal, pain, and decreased lubrication), menopause (hot flushes and sleep difficulties), and bowel/urinary issues. An electronic version for use at home via a patient portal or on an iPad at clinic was developed by our institution's Web Survey Core (Data Supplement). An automated clinical summary of a patient's responses prompts alerts for symptoms of concern and actionable responses (Fig 1). Before expansion of its use, the GST and clinical summary were tested with 10 patients with endometrial cancer (who underwent surgical intervention) in one attending's clinical practice for usability and administrative flow. The institutional review board approved implementation of the GST and the retrospective review of data collected.

### Implementation

The electronic tool was subsequently incorporated into use for patients with endometrial cancer at two clinics after clinical coordinators, gynecologic surgery attendings, advanced practice providers, nurses, and research staff received appropriate training. Newly diagnosed patients were told about the tool at their initial postoperative visit, and a letter of introduction was e-mailed at least one month before their first postoperative follow-up. Established patients currently being followed, who had completed surgery at least one year before the tool's implementation, were e-mailed a letter of introduction before their next clinical follow-up appointment.

Patients are selected to receive the Gynecologic Clinical Care survey on the basis of five primary factors: (1) a previous valid surgical procedure performed at Memorial Sloan Kettering Cancer Center (MSK); (2) a valid diagnosis; (3) an upcoming gynecologic appointment scheduled or completed from 7 days before; (4) no Gynecologic Clinical Care surveys submitted in MSK Engage within the previous 30 days; and (5) not deceased. All data and logic are run via a single script that queries the electronic health record (EHR). The cohort script is run nightly to capture new patients with characteristics that satisfy the above survey assignment conditions and to exclude patients who should no longer receive the survey (eg, if their upcoming appointment has since been canceled). The nightly list of valid patients is passed to the MSK Engage survey scheduler, which applies its own logic to ensure patients do not receive duplicate surveys for the same appointment event. The scheduler will then assign assessments, cancel assessments, and manage availability/expiry dates for assessments as needed.

The GST clinical summaries monitor symptoms, graph areas of concern, and prompt alerts for actionable responses (Fig 1). For example, if a patient is experiencing vaginal dryness, the platform activates an alert to the provider to discuss and provide patient education about vulvovaginal health. The PROs were obtained electronically using MSK Engage, a robust, user-friendly, and secure platform accessed through the MSK Portal. MSK Engage is a proprietary tool developed by Memorial Sloan Kettering Cancer Center. The tool is integrated with the EHR. Advanced visualization of PROs is created through a software extension Advanced Reports. Advanced Reports supports customized visualizations, algorithms, and statistical modeling to provide patients and clinicians with real-time analytics. Reports are available in MSK Engage and also inside the clinician documentation from a single sign-on. A copy of the report is sent to the EHR after being viewed by the clinician, and discrete data fields are stored in the data warehouse.

After a pilot period (12-24 months), the GST was expanded for use among patients with cervical cancer, and then rolled out to the entire Gynecology Service, Department of Surgery, at our institution (10 attendings during the study's time period).

A key component to implementation was training licensed practitioners (LIPs) to access the clinical symptom report generated upon patient submission of the GST. LIPs, including attending physicians, clinic nurses, advanced nurse practitioners, and physician assistants, were authorized to pull the automated clinical summary report into clinical documents. Clinical teams were asked to designate one team member responsible for pulling patient clinical summary reports into clinical documents and for providing patient education or referrals for the presenting symptoms. The clinical summary report is automatically stored by MSK Engage in the patient EHR once it is pulled into the LIP's clinical document.

### Study Cohort

Patients were assigned the GST surveys on the basis of surgical code, which identified (1) newly diagnosed patients, to be invited preoperatively to participate in the intervention, and (2) established patients who had already undergone surgery at least one year before, to be invited to participate. The first group (newly diagnosed) provides information on symptoms that may emerge within the short-term postoperative period; the second group (established) provides information about symptoms that may persist into long-term survivorship. The second group also represents patients who did not participate in the intervention in the immediate postoperative period as a means of identifying and managing symptoms. The data for these two groups (newly diagnosed and established) are presented separately.

### Statistical Analysis

Among newly diagnosed patients who completed a GST survey from the start of their postoperative follow-up period, we sought to determine whether there was an association between patient characteristics and the number of surveys completed. To this end, we used separate negative binomial regression models with number of surveys completed as the outcome, and each of the characteristics as the predictor. As patients with longer follow-up would have the opportunity to answer more surveys, we also incorporated the amount of follow-up into the model as the offset parameter. There was no evidence of overdispersion; final results are from Poisson regression models.

For our primary analysis, we examined the change in rates for symptoms reported over time. To this end, among newly diagnosed patients who completed the assessment, we used longitudinal models using generalized estimating equations with an exchangeable correlation structure. Each symptom was dichotomized to any symptom or severity (*v* no symptom or no severity). We then visualized the relationships by plotting the predicted probability of having that symptom, calculated from the respective generalized estimating equations models, against the survey time point (immediate postoperative period and 3-month, 6-month, and 12-month periods). All statistical analyses were conducted using R 4.0.1.

## RESULTS

We identified 3,357 GST assessments from MSK Engage assigned between January 1, 2019, and February 29, 2020, to 1,405 women who underwent surgery for endometrial or cervical cancer on or after January 2016. The tool demonstrated feasibility, with a 71% (2,370/3,357) completion rate of assigned tool assessments; 82% of the patients completed at least one assessment. Sixty-eight percent of assessments (1,612/2,370) were completed at home via the patient portal and 32% at follow-up appointments with a clinic iPad. Time for completion was minimal, with a median of 2 minutes (interquartile range, 1-3 minutes); 90% of patients completed the assessment within 5 minutes.

Among the 2,370 surveys completed, we excluded nine that were duplicates. We then grouped surveys on the basis of the postoperative time period in which they were answered: immediate postoperative period (< 2 months after surgery), 3-month period (2-5 months after surgery), 6-month period (5-8 months after surgery), 1-year period (8-14 months after surgery), 18-month period (14-20 months after surgery), 2-year period (20-28 months after surgery), and 3-year period (32-40 months after surgery). One hundred forty-one surveys that were not completed within any of the above time periods were excluded. Two hundred seventeen surveys were completed by established patients who underwent surgery before implementation of the GST; their short-term follow-ups after surgery were excluded, as they were not completed in the immediate postoperative period. The final data set consisted of 1,591 surveys completed by 1,051 patients, 485 of whom were followed with the surveys after surgery; the remaining 566 were established patients completing the surveys on long-term survivorship.

### Sample Characteristics and Assessments

Patient characteristics, stratified by follow-up group, for the 1,051 women who were diagnosed and treated for endometrial (n = 912) or cervical cancer (n = 139) and answered at least one survey are shown in Table 1. The majority were White (76%), married/partnered (62%), and had undergone a laparoscopic procedure (88%).

**TABLE 1.**
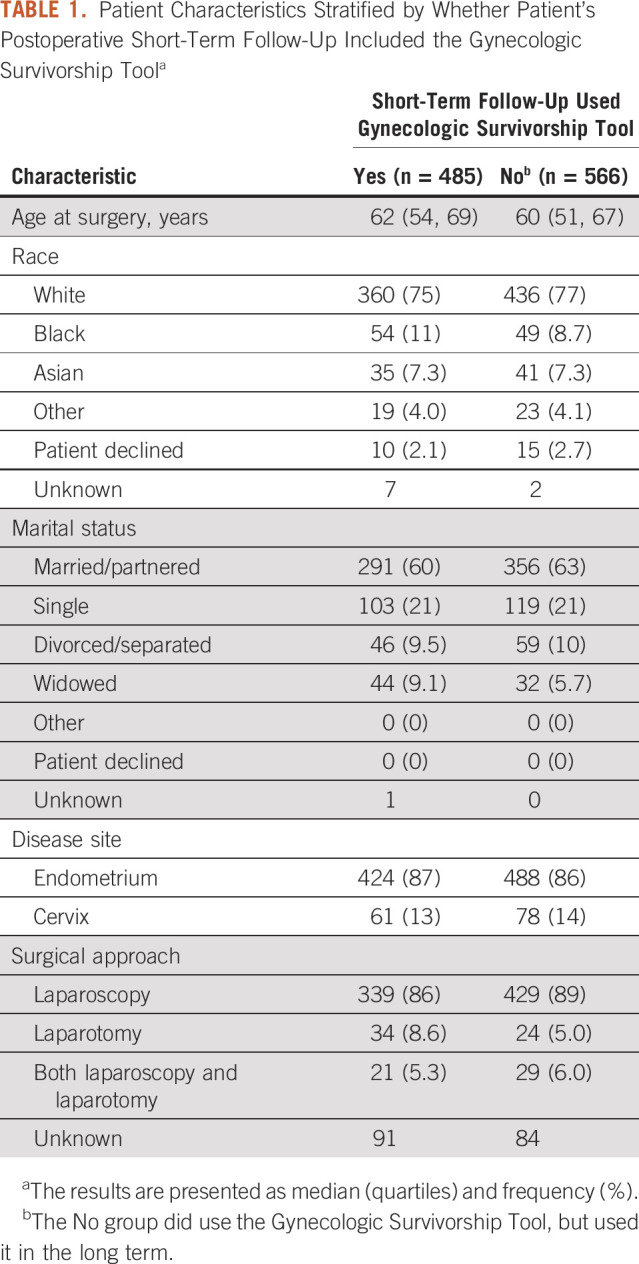
Patient Characteristics Stratified by Whether Patient's Postoperative Short-Term Follow-Up Included the Gynecologic Survivorship Tool^a^

The results assessing the association between characteristics and number of completed surveys are presented in Table 2. We found a significant association between older age and fewer surveys completed (incidence rate ratio [IRR] per 10 years of age, 0.94; 95% CI, 0.92 to 0.96; *P* < .001). For example, for two patients with the same amount of follow-up, the predicted number of surveys completed by a 35-year-old was 2.25 compared with 1.82 for a 70-year-old. We found evidence of an association between marital status and number of surveys completed: widowed patients completed fewer surveys compared with married/partnered patients (predicted number of surveys, 1.61 *v* 2.00; IRR, 0.80; 95% CI, 0.72 to 0.90; *P* < .001). Patients with cervical cancer (compared with endometrial cancer) and those who had undergone laparotomy (compared with laparoscopy) completed more surveys (predicted number of surveys, 2.30 *v* 1.89; IRR, 1.22; 95% CI, 1.13 to 1.31; *P* < .001; and predicted number of surveys, 2.38 *v* 1.86; IRR, 1.28; 95% CI, 1.16 to 1.40; *P* < .001, respectively).

**TABLE 2.**
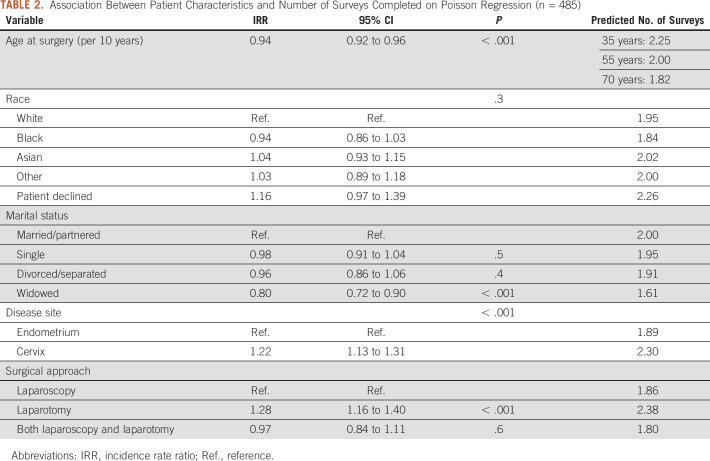
Association Between Patient Characteristics and Number of Surveys Completed on Poisson Regression (n = 485)

### Gynecologic Cancer Survivorship Symptoms

The results from the respective generalized estimating equations model for each of the symptoms on longitudinal analysis are shown in Table 3. We found evidence of changes in reported symptoms in newly diagnosed patients for the following: bowel habit problems, swelling or fluid, pain during examination, vaginal dryness, vulvar dryness, and vaginal bleeding (all *P* ≤ .023; Table 3). The observed probabilities of each symptom at each time period among established patients completing the GST in long-term survivorship are presented in Table 4. For some women, problems with bowel function (41%; 95% CI, 31 to 50), urinary function (31%; 95% CI, 22 to 40), insomnia (35%; 95% CI, 26 to 45), and hot flashes (24%; 95% CI, 17 to 34) persisted until the 3-year period after surgery. The probability of pain on examination and vaginal bleeding were low at the 3-year period (6%; 95% CI, 2 to 12; and 5%; 95% CI, 2 to 11, respectively), indicating that some women need long-term intervention.

**TABLE 3.**
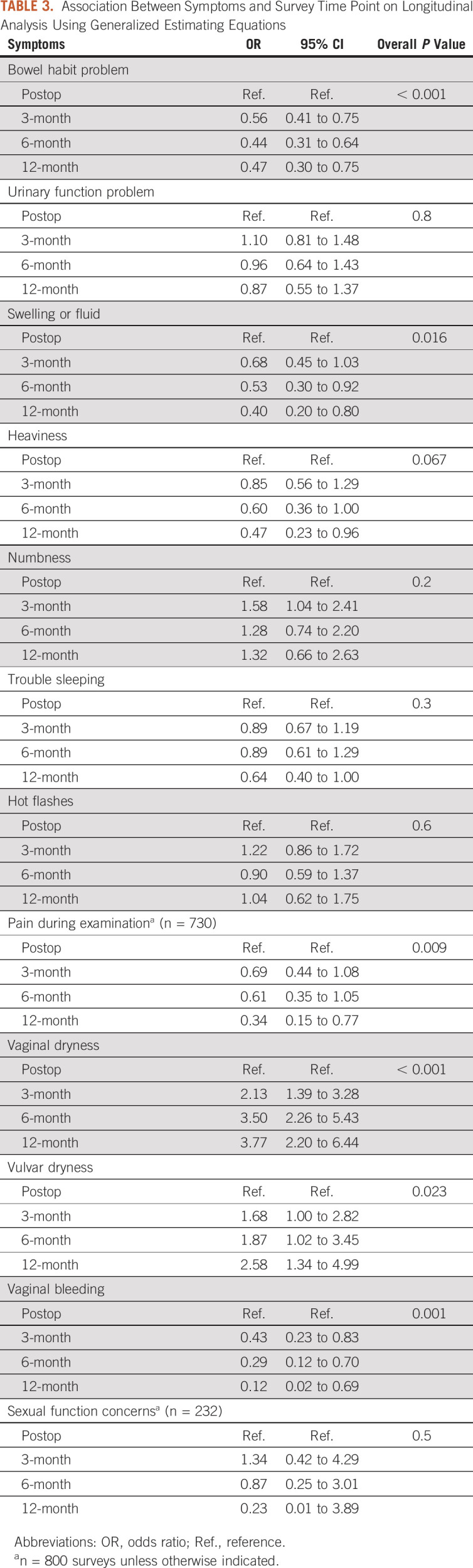
Association Between Symptoms and Survey Time Point on Longitudinal Analysis Using Generalized Estimating Equations

**TABLE 4.**
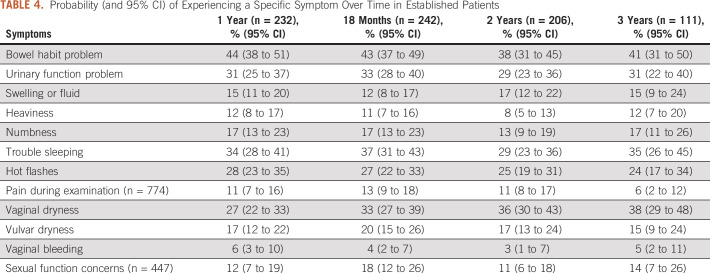
Probability (and 95% CI) of Experiencing a Specific Symptom Over Time in Established Patients

### Implementation Challenges

We faced several challenges after implementing the tool into the clinical setting. These included screening for patients who did not complete assessments at home, developing a standardized workflow among multiple clinical teams, and ensuring that all assessments were reviewed and entered into the medical record system. To address these issues, we created a multifaceted approach that incorporated a screening system and provided multidisciplinary feedback and training sessions across disciplines. Daily screening e-mails were sent to clinic coordinators to flag patients who completed or needed to complete the assessment. Printed copies of the GST clinical symptom reports were provided to the clinical team to facilitate discussion and assist with documentation. Educational meetings were conducted with administration, nursing, advanced practice providers, and attending staff. Weekly compliance updates were sent to the medical teams. With these initiatives, compliance improved within the first 8 months of implementation.

## DISCUSSION

The GST was found to be a feasible digital health tool for patients with gynecologic cancer, with good completion rates and minimal time commitment. The goal was to identify patients with survivorship symptoms and integrate this information into the clinical setting with a clinical summary using targeted questions to reduce patient burden and enhance participation, so that these issues would be addressed and monitored throughout the continuum of care.

Training and inclusion of all members of the administration and clinical team, as well as our multifaceted approach incorporating a screening system, and multidisciplinary feedback, were crucial to the implementation process. Daily screening e-mails were sent to clinic coordinators to flag patients assigned to the GST assessment. Feedback from nurses and clinic coordinators was elicited to address specific implementation challenges. Preferences that varied per clinical team included printing hard copies versus screen reading of the clinical summary report, documentation methods, and level of involvement of team members (nursing, advanced practice providers, and physicians). Providing physicians with paper-based reminders during office visits has been shown to be an effective strategy for improving compliance.^6^ This was the case with our survivorship tool. The visual reminders enhanced discussions and facilitated clinical flow and documentation. Quick guides about printing clinical summaries, access to electronic reports, and methods for submitting documentation were developed for the administrative/clinical team.

Clinic compliance also improved with LIP and administrative staff education sessions. Education sessions provided the team members the opportunity to discuss their experiences and share efficient methods for implementation of the tool in a busy clinical setting. LIPs received weekly compliance updates with details on the number of assessments completed and the number of reports reviewed and submitted in each clinic.

Research suggests that the most common long-term symptoms unique to gynecologic cancer survivors are lymphedema, urinary dysfunction, bowel problems, and pelvic pain.^12^ A recent Gynecologic Oncology Group study assessing lower-extremity lymphedema in patients with gynecologic cancer found that leg volume increases peaked at 6 weeks postoperatively and persisted for over 2 years of follow-up.^13^ We recognize the goal of cancer surveillance appointments is to ensure that cancer is not present. As such, survivorship symptoms and concerns may not be routinely discussed. Patients may also be uncomfortable raising their symptom concerns (eg, sexual health). However, discussion of sexual health concerns early in the treatment trajectory can lead to better outcomes and quality of life.^14^ Electronic platforms provide an opportunity for patients and clinicians to track and manage postoperative symptoms outside of the busy clinical setting. Screening e-mails and customized paper printouts alerted clinicians to review postoperative symptoms as a part of clinic preparation, before seeing the patient. The GST appears to be a feasible, time-saving, and user-friendly method that formalizes the routine screening of patients' bothersome symptoms so that these symptoms can be addressed at upcoming follow-up visits.

The inclusion of newly diagnosed as well as established patients already undergoing treatment allowed us to capture short- and long-term symptoms. These have the potential to negatively affect quality of life and intimacy; if these are identified in a timely way, however, simple intervention strategies such as medications and moisturizers for vaginal/vulvar health could provide symptom relief. Constipation could be addressed with increased hydration, stool softeners, and/or dietary modifications. Incontinence could be addressed with pelvic floor physical therapy or consultation with a urologist.

In future studies, we will examine more in-depth longitudinal data and compare symptoms reported between patients with cervical and endometrial cancer. Ultimately, the GST was designed and implemented to ensure that the symptoms and concerns of survivorship are not overlooked, even within busy clinics, and that these women receive the timely interventions they need. On the basis of the challenges that we encountered regarding clinical workflow, a direct patient interactive tool could be ideal for symptom management outside of the busy clinical setting. A patient interactive tool could provide information about self-management for mild or moderate symptoms directly to the patient as well as alert the clinical team via e-mail in case of severe symptoms reports. Tools that target individual supportive care needs have been shown to enable patient participation in their own care and reduce symptom burden.^15-18^ We hope to modify the GST in the near future so that personalized symptom management information can be communicated directly to the patient using the electronic platform, with detailed alerts to discuss these with the medical team at her next follow-up appointment.

In conclusion, in patients with gynecologic cancer, attention to sexual health concerns early in treatment and follow-up can lead to better outcomes and quality of life. The GST is a user-friendly and effective digital tool, requiring minimal time commitment. Electronic platforms take symptom data and create meaningful ways for patients and clinicians to track postoperative symptom concerns virtually outside of the clinical setting, enabling the medical team to develop strategies for addressing patients' symptoms and target concerns at follow-up. Health care systems provide an important opportunity for safe, time-efficient, and effective delivery of care, and should be considered as a potential investment by institutions.
